# Long non-coding RNA TGFB2-OT1 as a diagnostic biomarker and ceRNA regulator in rheumatoid arthritis

**DOI:** 10.3389/fgene.2026.1752878

**Published:** 2026-03-18

**Authors:** Yuqun Wang, Junhong Liu, Linping Du, Xiaodong Wang, Yuhua Su

**Affiliations:** 1 Department of Rheumatology and Immunology, Shandong Province Rheumatic Disease Key Speciality, Affiliated Hospital of Shandong Second Medical University, Weifang, China; 2 Key Laboratory of Rheumatology and Immunology, Weifang, China; 3 School of Public Health, Shandong Second Medical University, Weifang, China; 4 Department of Clinical Research Center, Affiliated Hospital of Shandong Second Medical University, Weifang, China

**Keywords:** bioinformatics, biomarker, E2F2, long non-coding RNA, rheumatoid arthritis, TGFB2-OT1

## Abstract

**Objective:**

Rheumatoid arthritis (RA) is a chronic systemic autoimmune disease characterized by persistent synovial inflammation and progressive joint destruction. Growing evidence highlights the critical role of lncRNAs in RA initiation and progression. However, the pathogenic contributions of many lncRNAs remain unclear.

**Methods:**

Whole-transcriptome sequencing of PBMCs from 5 RA patients and 5 healthy controls identified differentially expressed lncRNAs. Candidate lncRNAs, selected by fold-change and expression level, were validated via qRT-PCR in an expanded cohort (56 RA, 18 SLE, 20 pSS, and 39 HCs). Diagnostic performance was assessed by ROC analysis, and bioinformatic predictions explored potential miRNA–mRNA–protein interactions and functional mechanisms of lncRNAs.

**Results:**

A study identified 2,162 differentially expressed lncRNAs, with 1,212 upregulated and 950 downregulated. Six lncRNAs with notable expression changes were chosen for qRT-PCR validation. TGFB2-OT1(NR_125715.1) and ENST00000413791 were significantly altered in RA PBMCs, with NR_125715.1 showing high diagnostic accuracy (AUC = 0.8610) and RA-specific expression. NR_125715.1 expression correlated positively with rheumatoid factor (r = 0.297, p = 0.036) and anti-cyclic citrullinated peptide antibodies (r = 0.3809, p = 0.0041). Bioinformatics suggested NR_125715.1 might act as a ceRNA regulating E2F2 via miR-6756-3p and interact with the FUS protein, affecting RNA metabolism and inflammatory signaling. No m6A methylation or CpG islands were found.

**Conclusion:**

NR_125715.1 shows RA-associated dysregulation in PBMCs and demonstrates diagnostic discrimination in our cohort. Bioinformatic analyses suggest that NR_125715.1 may participate in RA-related regulatory programs, potentially involving a ceRNA axis (miR-6756-3p/E2F2) and a predicted interaction with the RNA-binding protein FUS. These mechanistic inferences are hypothesis-generating and require functional validation in future studies.

## 1 Introduction

Rheumatoid arthritis (RA) is a chronic systemic inflammatory autoimmune disorder characterized by persistent synovitis and progressive destruction of bone and articular cartilage, which frequently leads to joint deformity and functional impairment. Although the global prevalence of RA has shown a declining trend, recent estimates indicate a decrease from 0.46% to 0.27%, both genetic and environmental factors are recognized as key contributors to its pathogenesis (Finckh et al., 2022). Moreover, a growing body of evidence underscores the significance of epigenetic mechanisms—such as non-coding RNA regulation, DNA methylation, RNA methylation, and histone modifications—in the pathogenesis and progression of RA (Krishna Priya et al., 2025; Sadeghi and Mokhtari, 2026).

Advances in bioinformatics and high-throughput sequencing have enabled the identification of numerous epigenetic regulators across different tissues, such as microRNAs (miRNAs), long non-coding RNAs (lncRNAs), and circular RNAs (circRNAs) (Huang et al., 2021). Among these, lncRNAs have garnered increasing attention in the field of autoimmune diseases. Defined as RNA transcripts longer than 200 nucleotides, lncRNAs are localized in either the nucleus or cytoplasm (Wen et al., 2025). Initially regarded as transcriptional “noise” or non-functional byproducts of RNA polymerase II (Costa et al., 2025), lncRNAs are now known to play complex and diverse biological roles. They participate in the regulation of DNA synthesis, transcription, and translation, and are involved in critical processes such as apoptosis, cell differentiation, proliferation, and the pathogenesis of various diseases, including inflammatory and autoimmune conditions (Mattick et al., 2023; Cheng et al., 2023). In the immune system, lncRNAs have emerged as key modulators of gene expression (Majeed et al., 2025). Numerous studies have implicated lncRNAs in RA pathogenesis, indicating that their abnormal expression, whether through upregulation or downregulation, may be closely associated with inflammatory activation, synovial cell apoptosis, oxidative stress, and the dysregulation of signaling pathways (Chatzopoulos et al., 2025). These findings suggest that lncRNAs hold promise as potential biomarkers and therapeutic targets in RA.

In this study, we employed whole-transcriptome sequencing to profile lncRNA expression in peripheral blood mononuclear cells (PBMCs) from RA patients and healthy controls. We identified significantly dysregulated lncRNAs as candidate genes, which were subsequently validated via quantitative PCR. The diagnostic utility of these lncRNAs was assessed using receiver operating characteristic (ROC) curve analysis. Additionally, we evaluated the specificity of candidate lncRNAs across multiple autoimmune diseases and examined their correlation with clinical indicators in RA patients. Finally, we utilized bioinformatic tools to predict the potential regulatory mechanisms of these lncRNAs and conducted preliminary experimental validation. Our aim is to elucidate the role of lncRNAs in RA and explore their potential as diagnostic biomarkers and therapeutic targets.

## 2 Materials and methods

### 2.1 Patients

This study recruited 61 rheumatoid arthritis (RA) patients from the Department of Rheumatology and Immunology at the Affiliated Hospital of Weifang Medical University between January 2022 and March 2023. For comparison, we enrolled 39 gender- and age-matched healthy controls (HC), along with 18 systemic lupus erythematosus (SLE) patients and 20 primary Sjögren’s syndrome (pSS) patients during the same period. The four groups were comparable in terms of gender, ethnicity, smoking status, alcohol consumption, and regional distribution. Detailed inclusion and exclusion criteria are provided in Supplementary Table 1.

The study protocol was approved by the Ethics Committee of the Affiliated Hospital of Weifang Medical University (Approval No: wyfy-2023-ky-022). All participants provided written informed consent for publication of their data. Demographic and clinical characteristics of the participants are summarized in Supplementary Table 2.

### 2.2 Isolation of peripheral blood mononuclear cell

Fasting venous blood samples (5 mL) were collected from RA, pSS, SLE patients and HCs using EDTA-K2 anticoagulant tubes. Samples were temporarily stored at 4 °C and processed within 2 h. PBMCs were isolated by Ficoll density gradient centrifugation. The harvested cell pellets were lysed with 1 mL TRIzol reagent (VICMED, China), incubated at room temperature for 5 min, and then stored at −80 °C for subsequent RNA extraction.

### 2.3 Whole transcriptome high-throughput sequencing

PBMC samples from 5 RA patients and 5 HCs were selected for whole transcriptome sequencing, which was performed by Shanghai Ouyi Biomedical Technology Co.

### 2.4 Reverse transcription and quantitative real-time PCR

RNA extraction, reverse transcription, and quantitative real-time PCR (qRT-PCR) were performed as previously described (Chatzopoulos et al., 2025). All primers were synthesized by Shanghai Sangong Bioengineering Co., Ltd., and their sequences are listed in Supplementary Table 3.

### 2.5 Analysis of lncRNA biological functions

Gene Ontology (GO) and Kyoto Encyclopedia of Genes and Genomes (KEGG) pathway enrichment analyses were performed using OECloud tools (https://cloud.oebiotech.com). GO terms covering biological processes, molecular functions, and cellular components were considered significant at P < 0.05. KEGG annotation was applied to uncover potential biological roles of differentially expressed genes.

MicroRNAs potentially binding to TGFB2-OT1(NR_125715.1) were predicted using the miRDB database (Chen and Wang, 2020) with a target score threshold > 85. Their functional roles were analyzed using DIANA-miRPath v3.0 (Vlachos et al., 2015). Prediction of relevant mRNAs using miRDB (Chen and Wang, 2020), miRTarBase (Huang et al., 2022), miRWalk (Sticht et al., 2018), RNA22 (Miranda et al., 2006), RNAInter (Kang et al., 2022), TargetMiner (Wen et al., 2018), TargetScan databases (McGeary et al., 2019), and construction of lncRNA-miRNA-mRNA ceRNA network.

Protein interactions with lncRNAs were predicted using catRAPID omics v2.0 (Agostini et al., 2013), RBPDB (Gerstberger et al., 2014), and AnnoLnc2 (Ke et al., 2020). Functional annotation of the predicted proteins was performed with Metascape (Zhou et al., 2019). Transcription factors regulating candidate protein-related genes were identified using the Cistrome DB database (Zheng et al., 2019).

RNA secondary structures were predicted using ViennaRNA Web Services (Gruber et al., 2015). Potential m6A methylation sites were predicted with the SRAMP online database (Fan et al., 2024), and CpG islands were detected using Emboss (Rice et al., 2000) and MethPrimer (Li and Dahiya, 2002).

### 2.6 Statistical methods

All statistical analyses were performed using GraphPad Prism 9.0 software. Data are presented as mean ± standard deviation. Group comparisons were conducted using Student’s t-test or one-way ANOVA, as appropriate. The correlation analysis between genes and clinical indicators was performed using Pearson’s correlation coefficient, while the correlation analysis between genes was conducted using Spearman’s correlation coefficient. Diagnostic performance of candidate lncRNAs was evaluated by ROC curve analysis, with the area under the curve (AUC) used as the evaluation metric. A P-value < 0.05 was considered statistically significant.

## 3 Results

### 3.1 Differentially expressed lncRNAs

**FIGURE 1:**
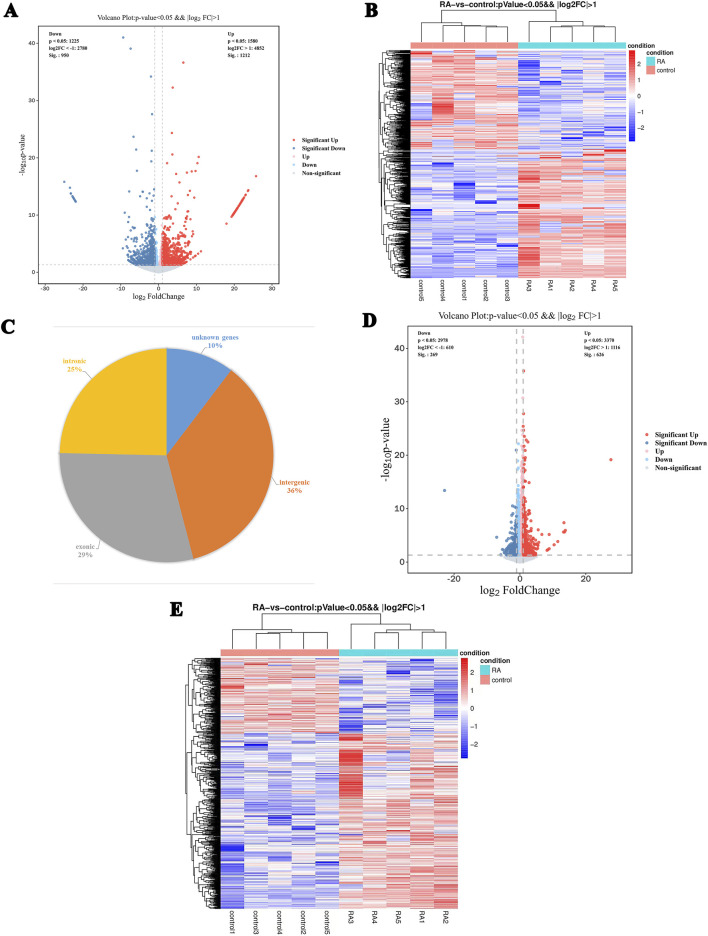
Characteristics of differentially expressed lncRNAs and mRNAs in PBMCs from RA patients and healthy controls. **(A)** Volcano plot of differentially expressed lncRNAs. Each dot represents one lncRNA, with red indicating significantly upregulated and blue indicating significantly downregulated lncRNAs. Gray dots represent non-significant transcripts. **(B)** Hierarchical clustering heatmap of the 2,162 differentially expressed lncRNAs. Red indicates high expression, blue indicates low expression. **(C)** Pie chart classification of differentially expressed lncRNAs based on genomic origin: intergenic (36%), exonic (29%), intronic (25%), and unannotated (10%). **(D)** Volcano plot of differentially expressed mRNAs. Red indicates upregulated mRNAs, blue indicates downregulated mRNAs. **(E)** Heatmap of differentially expressed mRNAs, with red representing high expression and blue representing low expression.

We profiled lncRNA expression in PBMCs obtained from five patients with RA and HCs using high-throughput RNA sequencing. In total, 16,433 distinct lncRNAs were detected. Of these, 2,162 lncRNAs were significantly differentially expressed (DE-lncRNAs) according to the criteria of |fold change| > 2 and p < 0.05, comprising 1,212 upregulated and 950 downregulated transcripts (Figure 1A). Unsupervised hierarchical clustering distinctly separated RA samples from HCs according to their lncRNA expression signatures (Figure 1B). Based on their genomic origin, DE-lncRNAs were classified into intergenic (36%), exonic (29%), intronic (25%), and unannotated (10%) categories (Figure 1C). In parallel, 895 differentially expressed mRNAs (p < 0.05, |fold change| ≥ 2) were identified, including 626 upregulated and 269 downregulated genes. Their expression profiles are illustrated in heatmap and volcano plot representations (Figures 1D,E).

### 3.2 Screening and validation of RA-associated lncRNAs

**FIGURE 2:**
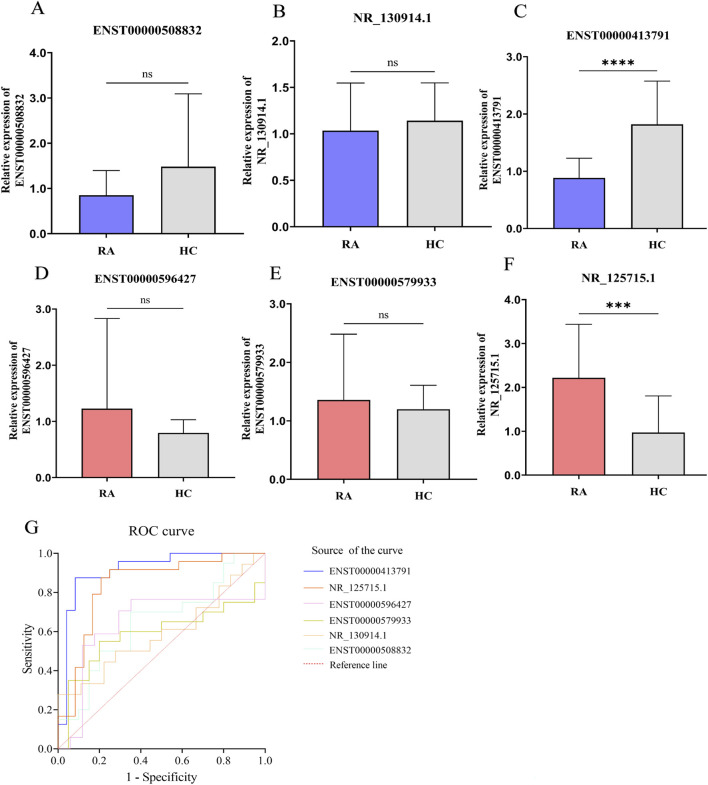
Screening and validation of differentially expressed lncRNAs in RA PBMCs. **(A-F)** Quantitative real-time PCR (qRT-PCR) validation of six candidate lncRNAs in PBMCs from 24 RA patients and 16 healthy controls (HCs). Expression levels are presented as 2^−ΔΔCT^ normalized to GAPDH. Bar graphs show mean ± SD; statistical significance was assessed by Student’s t-test. **(G)** Receiver operating characteristic (ROC) curve analysis of the six lncRNAs for distinguishing RA patients from HCs. Area under the curve (AUC) values are indicated. ns: not significant; ***: p < 0.001; ****: p < 0.0001.

To identify robust candidate lncRNAs, we applied stringent filtering criteria: (1) p < 0.05 and fold change (FC) > 2 or < 0.5; (2) average FPKM > 3 in at least one group; (3) average FPKM < 50 in at least one group; and (4) inclusion of annotated lncRNAs. Based on the magnitude of differential expression, three upregulated (ENST00000596427, ENST00000579933, NR_125715.1) and three downregulated (ENST00000508832, NR_130914.1, ENST00000413791) lncRNAs were selected for subsequent experimental validation (Table 1). Quantitative real-time PCR (qRT-PCR) confirmed the altered expression of all six candidates; however, only ENST00000413791 and NR_125715.1 exhibited statistically significant differences (Figures 2A–F). ROC curve analysis revealed strong diagnostic performance for ENST00000413791 and NR_125715.1, with AUC of 0.8906 (95% CI, 0.7742–1.000; p < 0.001) and 0.8385 (95% CI, 0.7179–0.9591; p < 0.001), respectively. The remaining four lncRNAs exhibited moderate diagnostic potential, with AUC values ranging from 0.58 to 0.65 (Figure 2G).

**TABLE 1:** Six differentially expressed lncRNAs selected for validation from RNA-seq data.

lncRNA_id	FoldChange	p-value	Regulation	Chromosome
ENST00000508832	0.03505386	2.71213E-05	Down	11
NR_130914.1	0.114045753	3.54E-12	Down	5
ENST00000413791	0.055006424	0.000329522	Down	2
ENST00000596427	45.99554489	2.01818E-16	Up	19
ENST00000579933	157.866962	3.04269E-10	Up	18
NR_125715.1	5.058254326	0.000215428	Up	1

### 3.3 NR_125715.1 as a novel potential biomarker for RA

**FIGURE 3:**
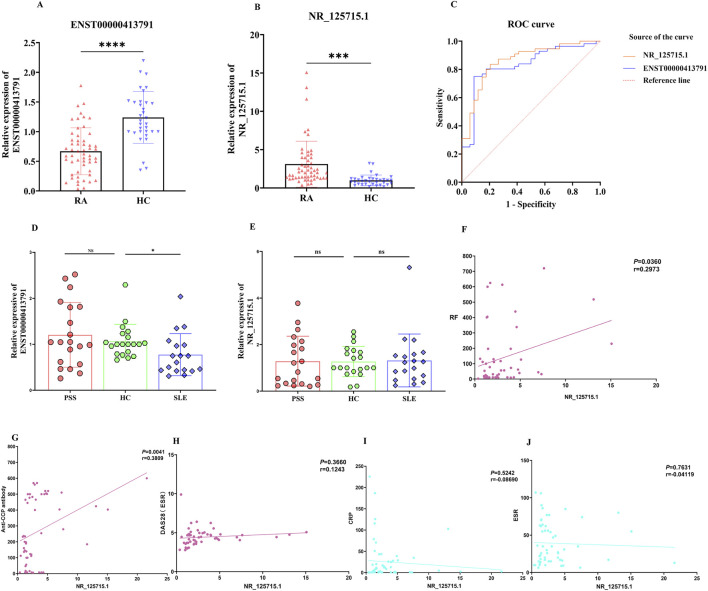
NR_125715.1 as a possible new potential diagnostic biomarker for RA and correlation with clinical features and laboratory tests. **(A,B)** Relative expression levels of ENST00000413791 and NR_125715.1 in patients with RA and HCs. Data are presented as 2^–ΔΔCT^ normalized to GAPDH. **(C)** ROC curve analysis of ENST00000413791 and NR_125715.1 in the expanded cohort. **(D,E)** Specificity analysis of lncRNA expression across disease groups (SLE, pSS) and HCs. **(F–J)** Correlation analysis between NR_125715.1 expression and clinical parameters: rheumatoid factor (RF), anti-cyclic citrullinated peptide (anti-CCP) antibody, Disease Activity Score 28 (DAS28), C-reactive protein (CRP), and erythrocyte sedimentation rate (ESR). Pearson’s correlation coefficient (r) and p-values are shown. ns, not significant; ***, p < 0.001; ****, p < 0.0001.

We further evaluated the diagnostic potential of ENST00000413791 and NR_125715.1 in an expanded cohort comprising 56 patients with RA, 18 with SLE, and 20 with pSS. Both lncRNAs remained significantly dysregulated in PBMCs from RA patients (Figures 3A,B). ROC curve analysis in this enlarged cohort yielded AUCs of 0.8304 for ENST00000413791 and 0.8610 for NR_125715.1 (Figure 3C). Clinically, these results support NR_125715.1 as a PBMC-based diagnostic biomarker candidate for RA classification; however, the present cross-sectional design does not establish prognostic value, longitudinal monitoring, or treatment-response utility. Specificity analysis revealed that ENST00000413791 expression was not significantly altered in pSS compared with HCs, whereas NR_125715.1 showed no significant difference in either pSS or SLE, supporting its RA-specific dysregulation (Figures 3D,E). We next examined correlations between NR_125715.1 expression levels and clinical parameters. NR_125715.1 expression levels showed a positive correlation with anti-CCP antibody titers (r = 0.3809, p = 0.0041) and rheumatoid factor (r = 0.297, p = 0.036), but not with ESR, CRP, or DAS28 scores (Figures 3F–J).

### 3.4 Potential role of NR_125715.1 in RA pathogenesis

Following the identification of NR_125715.1 as a promising diagnostic biomarker, we proceeded to investigate its potential functional mechanisms in RA. Since lncRNAs are known to function through diverse molecular mechanisms, such as signaling, decoy, guidance, and scaffolding functions (Wang et al., 2021), we performed comprehensive bioinformatic analyses to predict microRNAs, proteins, and coding genes that might interact with NR_125715.1.

#### 3.4.1 Potential miRNA sponge function of NR_125715.1

**FIGURE 4:**
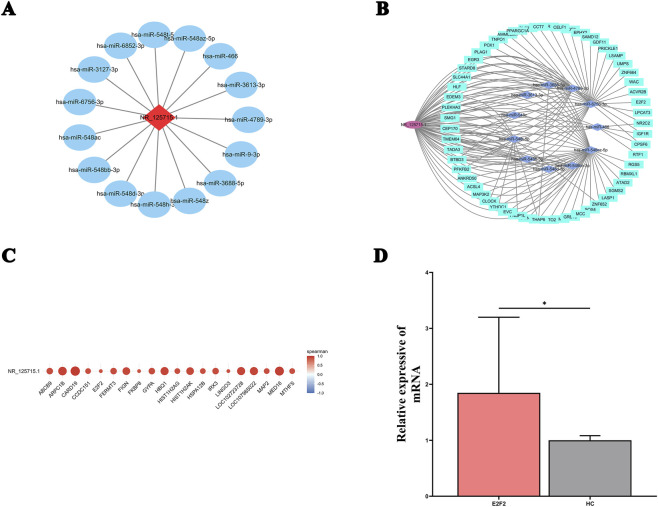
Prediction of NR_125715.1 miRNA sponge function and ceRNA network construction. **(A)** List of 15 miRNAs predicted to bind NR_125715.1 with high confidence (target score > 85) using the miRDB database. **(B)** Competing endogenous RNA (ceRNA) network of NR_125715.1–miRNA–mRNA interactions constructed using Cytoscape. Diamond node:lncRNA, Ellipse:miRNA, and Rectangle:mRNA. **(C)** Heatmap showing correlation between NR_125715.1 expression and 154 significantly correlated mRNAsfrom RNA-seq data. **(D)** Expression of candidate mRNAs in the RA. qRT-PCR was conducted on RNA in PBMCs from 20 RA patients and 14 HCs. Data are presented as 2^−ΔΔCT^ normalized to GAPDH expression.

We first investigated whether NR_125715.1 could act as a competitive endogenous RNA (ceRNA). Using the miRDB database, we identified 15 microRNAs (miRNAs) with high predicted binding potential, defined by a target score exceeding 85 (Figure 4A). Functional enrichment analysis using DIANA-miRPath v3.0 revealed that these miRNAs were involved in 92 Gene Ontology (GO) biological processes, mainly related to gene expression regulation, nucleic acid–binding transcription factor activity, and protein modification (Supplementary Figure S1). KEGG pathway analysis further demonstrated their enrichment in 39 signaling pathways, including Hippo, FoxO, Wnt, MAPK, Ras, and PI3K–Akt pathways, all of which are well recognized for their involvement in RA pathogenesis (Supplementary Figure S2). We subsequently predicted target mRNAs for these 15 miRNAs and identified 57 overlapping targets across multiple databases. These interactions were integrated to construct a comprehensive NR_125715.1–miRNA–mRNA ceRNA network using Cytoscape (Figure 4B). To independently validate these predictions, RNA-seq data were analyzed, revealing 154 mRNAs significantly correlated with NR_125715.1 expression (|r| > 0.8, p < 0.01; Figure 4C). Gene Set Enrichment Analysis (GSEA) of these correlated mRNAs demonstrated pathway enrichments largely consistent with those predicted by miRNA analysis, including axon guidance, MAPK, Rap1, PI3K–Akt, and cancer-related pathways (Supplementary Figure S3). By intersecting miRNA-predicted and expression-correlated mRNAs, E2F2 emerged as a particularly promising target gene, and qRT-PCR validation confirmed that E2F2 expression was significantly upregulated in RA PBMCs relative to HCs (Figure 4D).

#### 3.4.2 Protein binding capacity of NR_125715.1

**FIGURE 5:**
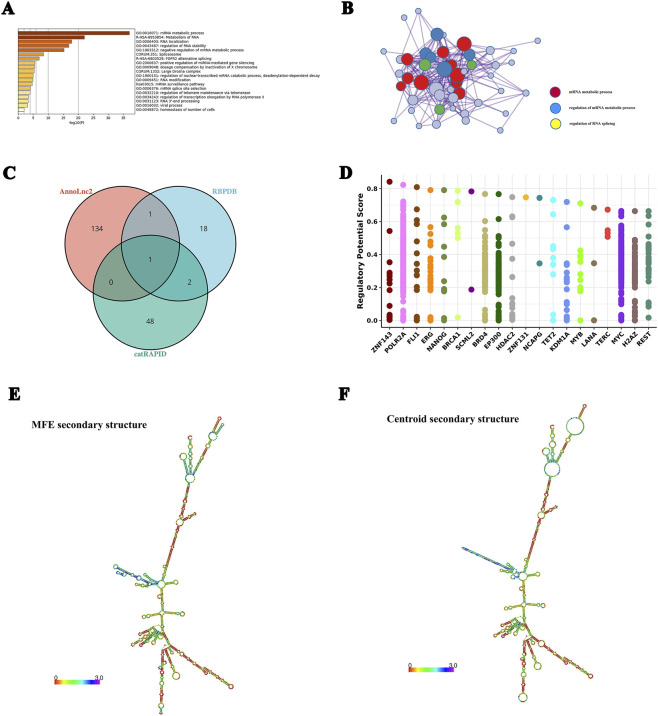
Functional enrichment and protein interaction prediction for NR_125715.1. **(A)** Gene Ontology (GO) enrichment analysis of 51 predicted NR_125715.1-binding proteins from catRAPID omics. **(B)** All protein-protein interactions 498 between genes form a PPI network. **(C)** Venn diagram showing overlap of predicted interacting proteins from catRAPID omics, AnnoLnc2, and RBPDB databases. FUS (P35637) was identified as a high-confidence interaction partner. **(D)** Predicted transcription factors regulating FUS expression, identified using Cistrome DB database. **(E,F)** Predicted secondary structure of NR_125715.1 using ViennaRNA Web Services, showing a stable stem–loop configuration potentially facilitating protein binding. ns: no significance *: p < 0.05; ****: p < 0.0001.

We further explored the protein-binding potential of NR_125715.1 through computational prediction. The catRAPID omics v2.0 platform predicted 51 potential interacting proteins, using a confidence score threshold above 2.4. Metascape enrichment analysis of these proteins revealed significant overrepresentation of biological processes associated with mRNA metabolism, RNA stability regulation, and positive regulation of miRNA-mediated gene silencing (Figure 5A). Protein–protein interaction (PPI) network analysis further implicated NR_125715.1 in mRNA metabolic regulation and RNA splicing processes (Figure 5B). To strengthen these predictions, data from multiple bioinformatic databases were integrated. The RBPDB database predicted 17 candidate RNA-binding proteins (RBPs), whereas AnnoLnc2 predicted 139 RBPs. Overlap analysis across catRAPID omics, AnnoLnc2, and RBPDB identified common predicted interacting proteins (Figure 5C). Among these, FUS emerged as the most promising binding protein. Using the Cistrome DB database, ZNF143, POLR2A, and FLI1 were identified as key transcription factors regulating FUS expression (Figure 5D). RNA secondary structure prediction revealed that NR_125715.1 forms a stable stem–loop configuration (Figures 5E,F), potentially facilitating interactions with binding proteins such as FUS.

#### 3.4.3 Possible special modifiers for NR_125715.1

To explore potential chemical modifications of NR_125715.1, we employed multiple computational prediction tools. The SRAMP online tool was used to predict potential m6A methylation sites, whereas Emboss CpGPlot and MethPrimer were applied to identify CpG islands within the NR_125715.1 sequence. No m6A methylation sites or CpG islands were detected within NR_125715.1 according to these analyses (Supplementary Figure S4).

## 4 Discussion

Rheumatoid arthritis is a systemic autoimmune disorder characterized by immune-mediated destruction of the synovial joint lining. Clinical symptoms typically include joint pain and swelling, most commonly in the hands and feet, which frequently progress to irreversible joint damage and functional disability. The disease affects approximately 0.5% of adults worldwide and exhibits a notable gender disparity, being two to three times more prevalent in women than in men (Gravallese et al., 2024). Although the precise etiology remains unclear, several risk factors have been established, including smoking, silica exposure, periodontitis, and genetic predisposition (Noto et al., 2025). In recent years, epigenetic mechanisms have gained increasing attention in RA research. Advances in molecular techniques have revealed that non-coding RNAs—including circular RNAs, long non-coding RNAs, and microRNAs—play crucial roles in gene regulation and the maintenance of physiological processes. Importantly, they also contribute significantly to the dysregulation of autoimmune and inflammatory responses (Tofigh et al., 2023). With the emergence of new transcriptomics technologies, the regulation of lncRNA transcription and processing has been studied in greater depth, and the transcriptional mechanisms involved in the potential functions of lncRNAs in diseases have contributed to the development of therapeutic tools for specific lncRNA transcript applications in personalized medicine (Nojima and Proudfoot, 2022). For example, one RNA sequencing study identified several aberrantly expressed lncRNAs related to apoptosis and autophagy in PBMCs from RA patients, suggesting their potential as diagnostic biomarkers (Wen et al., 2020). Similarly, Xu et al. (2023) reported that lncRNA PICSAR was markedly upregulated in RA fibroblast-like synoviocytes and synovial fluid, where it promotes synovial cell proliferation, migration, and inflammation via miR-4701-5p sponging. These findings collectively underscore the potential of lncRNAs as both biomarkers and functional regulators in RA.

### 4.1 Differential expression of lncRNAs in RA

In this study, we performed whole-transcriptome sequencing of PBMC samples from five RA patients and five healthy controls. We identified 2,162 differentially expressed lncRNAs and 895 differentially expressed mRNAs. Functional enrichment analysis indicated that these mRNAs are involved in immune and inflammatory responses, including IL-6 production and several inflammation-related signaling pathways. Notably, we identified six core genes—ALB, IL-10, MMP9, FN1, CXCL8, and TNF—that may participate in RA pathogenesis through immune-inflammatory responses, cytokine activity, and JAK-STAT signaling. Previous studies have suggested that lncRNAs may regulate such genes and pathways, thereby contributing to RA progression (Elhai et al., 2023; Li et al., 2022).

Using a multi-step screening strategy, we selected six candidate lncRNAs (three upregulated and three downregulated) for further validation. RT-qPCR confirmed that the expression trends of all six candidates were consistent with the sequencing data. Among them, ENST00000413791 and NR_125715.1 exhibited statistically significant differential expression. Several lncRNAs, including PICSAR (Bi et al., 2019), GAS5 (Cieśla et al., 2024),NEAT1 (Al Karamany et al., 2025), and IFNG-AS1 (Zhao et al., 2024) have previously been proposed as diagnostic biomarkers for RA. Our findings suggest that ENST00000413791 and NR_125715.1 may also serve as RA-specific diagnostic markers.

To further assess their clinical potential, we expanded the validation to a larger cohort that included patients with SLE and pSS. Both lncRNAs remained significantly dysregulated in RA patients. Notably, NR_125715.1 exhibited higher diagnostic accuracy than ENST00000413791, with an AUC of 0.8610 in the expanded cohort. Importantly, NR_125715.1 expression was not significantly altered in PBMCs from patients with SLE or pSS, indicating a high degree of disease specificity. In contrast, ENST00000413791 was also downregulated in SLE patients, limiting its specificity for RA.

NR_125715.1 correlated with RF and anti-CCP but showed no significant association with ESR, CRP, or DAS28 in this cohort. This pattern may indicate closer linkage to serological autoimmunity [autoantibody status (Steiner and Toes, 2024)] than to cross-sectional systemic inflammation or composite clinical activity indices, which can be affected by treatment and temporal variability. Larger prospective studies are needed to determine whether NR_125715.1 tracks longitudinal disease activity or treatment response.

NR_125715.1 was prioritized using a multi-step strategy integrating RNA-seq screening, qRT-PCR confirmation of consistent expression trends, and clinical utility metrics. Among candidate lncRNAs, NR_125715.1 demonstrated significant RA-specific dysregulation, higher diagnostic accuracy (AUC = 0.8610), and minimal alteration in SLE or pSS, supporting disease specificity. Given the limited prior functional characterization of NR_125715.1 in RA, we selected it for focused mechanistic prediction analyses.

From a clinical perspective, NR_125715.1 may be explored as an adjunct PBMC-based biomarker to support RA classification, particularly when standard serologies are negative or equivocal or when CTDs are part of the differential diagnosis. Importantly, our current evidence supports diagnostic discrimination within the tested groups, but does not establish prognostic value or longitudinal tracking. Because NR_125715.1 correlated with RF and anti-CCP, its independence and incremental value beyond established serologies should be tested using multivariable models and model-comparison metrics (e.g., delta AUC/DeLong test and reclassification indices) in larger prospective cohorts.

### 4.2 Potential biological functions of lncRNA

To explore the functional role of NR_125715.1 in RA, we performed a series of bioinformatic analyses. We first hypothesized that it may act as a competitive endogenous RNA. Using the miRDB database, we identified 15 microRNAs with high binding potential to NR_125715.1. Enrichment analysis indicated that these miRNAs are involved in gene expression, transcription factor activity, and protein modification, and are linked to several signaling pathways such as Ras, PI3K-Akt, and Wnt. By integrating miRNA target predictions with mRNA expression data obtained from RNA sequencing, we developed a ceRNA network and identified E2F2 as a critical downstream target. Previous studies have implicated E2F2 in the regulation of inflammatory responses in RA (Zhang et al., 2018; Xu et al., 2021). Experimental validation demonstrated a significant upregulation of E2F2 in PBMCs from RA patients, supporting the hypothesis that NR_125715.1 may act as a ceRNA by sequestering miRNAs such as miR-6756-3p. This sequestration potentially elevates E2F2 expression, thereby contributing to RA-associated inflammation. Further research has shown that E2F2 is overexpressed in RA synovial tissue, where it participates in the pathogenesis and progression of RA by modulating biological processes such as metabolism, stress responses, and ribosomal synthesis in RA synovial fibroblasts (Steiner and Toes, 2024). Additionally, cytokines such as interleukin-6 (IL-6) and tumor necrosis factor-alpha (TNF-α) can induce E2F2 expression in RA synovial fibroblasts through distinct signaling pathways (Chang et al., 2014).

In addition to its miRNA sponge function, NR_125715.1 may also interact directly with proteins. We identified 51 candidate binding proteins using catRAPID omics, and functional enrichment analysis suggested their involvement in mRNA metabolic processes and RNA stability regulation. By integrating predictions from RBPDB and AnnoLnc2, we identified FUS (P35637) as a high-confidence interaction partner. FUS has been studied in various pathological contexts; for example, it has been linked to mitochondrial dysfunction and cardiomyocyte death (Wang et al., 2022), and shown to activate the IL-6/STAT3 signaling pathway via miR-181a-5p (Zhao et al., 2022). RNA secondary structure prediction indicated that NR_125715.1 forms a stable stem-loop structure, which may facilitate its binding to FUS. We further identified ZNF143, POLR2A, and FLI1 as transcription factors regulating FUS expression. Based on these findings, we speculate that NR_125715.1 may influence RA pathogenesis through direct interaction with FUS or cooperative regulation of FUS-related pathways.

Finally, we examined whether NR_125715.1 undergoes common post-transcriptional modifications such as m6A methylation or CpG island methylation, both of which can influence lncRNA stability, localization, and function. However, bioinformatic screening using SRAMP, Emboss, and MethPrimer did not identify m6A sites or CpG islands within the NR_125715.1 sequence.

In summary, our findings highlight the strong potential of NR_125715.1 as a diagnostic biomarker for RA. Further investigation is needed to determine whether its functional effects are mediated primarily through ceRNA mechanisms or protein interactions. Elucidating these mechanisms may not only deepen our understanding of RA pathogenesis but also provide a foundation for future targeted therapies.

## 5 Conclusion and future directions

In this study, we performed comprehensive transcriptomic profiling of PBMCs from RA patients and identified NR_125715.1 as a novel, RA-specific lncRNA. Its expression positively correlated with anti-CCP antibodies and rheumatoid factor, indicating a close relationship with disease activity and autoantibody production. Mechanistically, bioinformatic analyses revealed that NR_125715.1 may function as a miRNA sponge regulating E2F2 expression via the miR-6756-3p/E2F2 axis, and may also bind to FUS, an RNA-binding protein implicated in RNA metabolism and inflammation. These dual regulatory roles suggest that NR_125715.1 could serve as a pivotal mediator of immune dysregulation in RA.

Despite these promising findings, several limitations should be acknowledged. The RNA-seq discovery cohort was small (5 RA vs. 5 HCs), and mechanistic inferences (ceRNA and protein-binding predictions) are hypothesis-generating. Although qRT-PCR supported RA-associated dysregulation of NR_125715.1 and upregulation of E2F2, direct regulation and binding require functional validation (e.g., luciferase reporter assays, perturbation-rescue designs, and RIP/CLIP assays). Future multi-center, prospective cohorts with broader disease controls and tissue-level validation (including synovium) are needed to establish clinical utility and biological generalizability.

Collectively, our findings expand the understanding of lncRNA-mediated regulation in RA and provide a foundation for developing lncRNA-based diagnostic biomarkers and precision therapeutic strategies for autoimmune diseases.

## Data Availability

The datasets presented in this article are not readily available because of ethical and privacy restrictions. Requests to access the datasets should be directed to the corresponding author.
