# Supplementary material for: Long non-coding RNA TGFB2-OT1 as a diagnostic biomarker and ceRNA regulator in rheumatoid arthritis
Source: Front Genet. 2026 Mar 18;17:1752878. doi: 10.3389/fgene.2026.1752878 (PMC13038230; doi:10.3389/fgene.2026.1752878)
Supplement: Supplementary file 1 [file DataSheet1.pdf]

Supplementary Material

1 Supplementary Figures

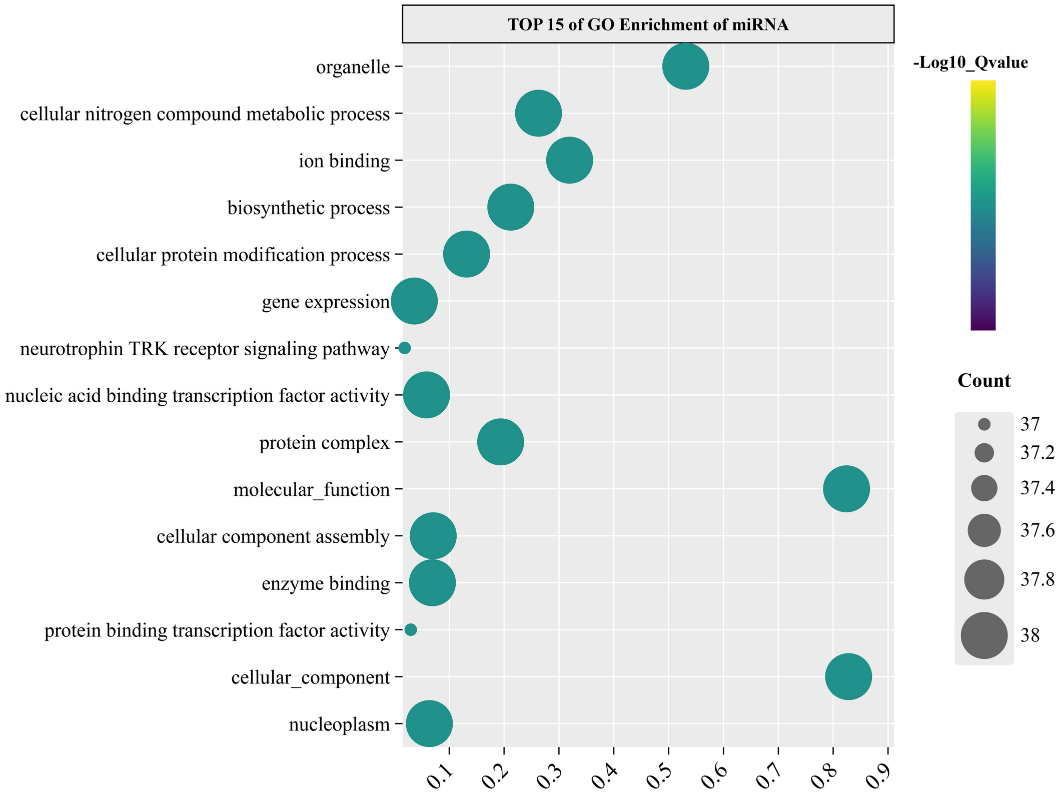

Supplementary Figure 1. The bubble chart illustrates the results of Gene Ontology analyses, along with disease prediction data.

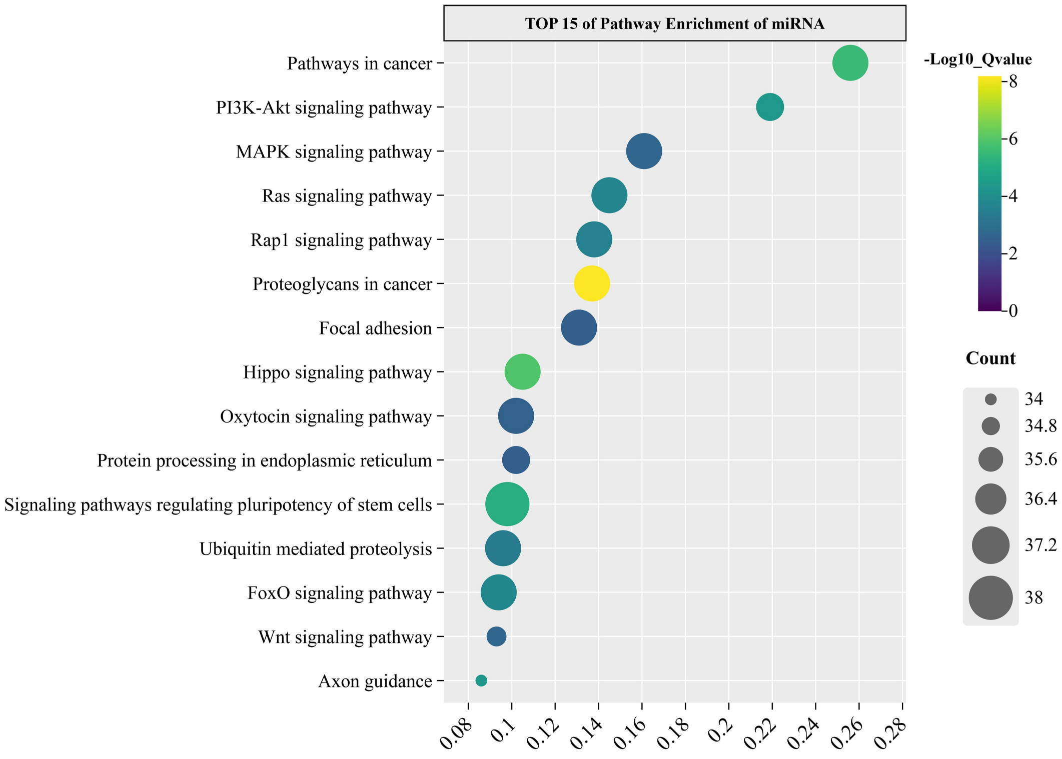

Supplementary Figure 2. The bubble chart illustrates the results of KEGG pathway analyses, along with disease prediction data.

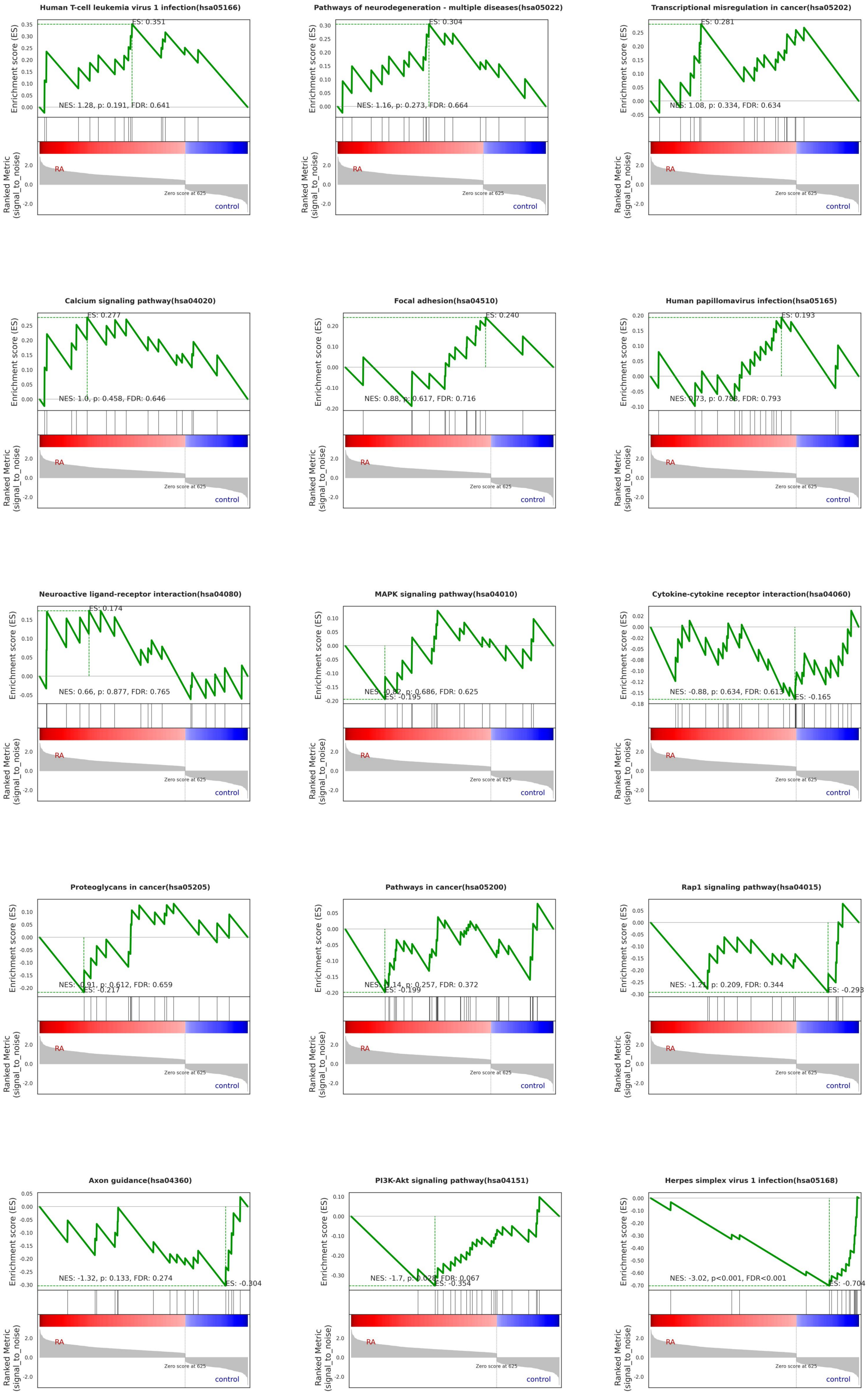

Supplementary Figure 3. GSEA Analysis of NR_125715.1-Related mRNAs.

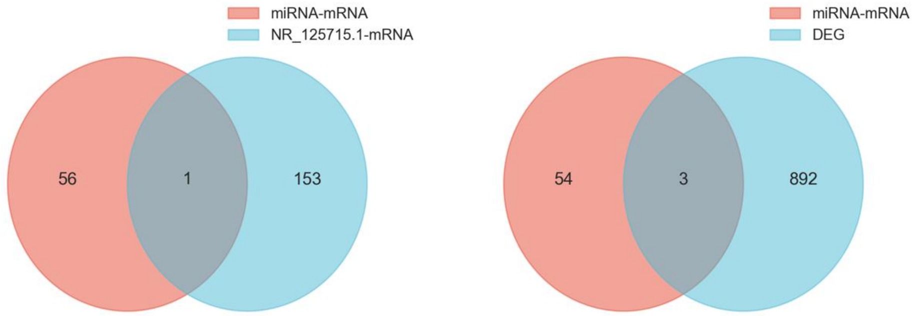

Supplementary Figure 4. Venn diagrams illustrate the overlap between predicted miRNAs and differentially expressed genes, as well as the overlap between predicted miRNAs and NR1235-associated mRNAs.

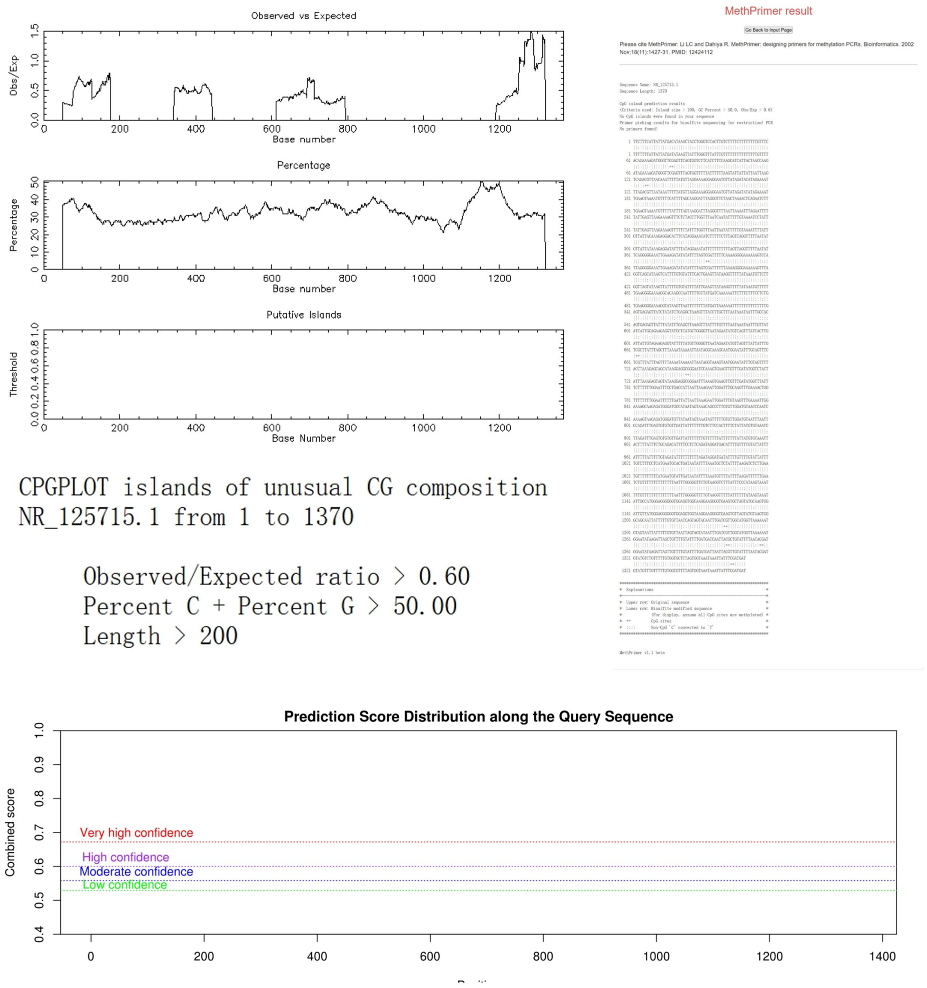

Supplementary Figure 5. Prediction of m6A methylation sites and CpG islands for NR_125715.1.

2 Supplementary Table

Supplementary Table 1 Sample Inclusion & Exclusion Criteria

Inclusion criteria	Exclusion criteria
the revised criteria for the classification of SLE published jointly by the European League Against Rheumatism (EULAR)/American College of Rheumatology (ACR) in 2019	Patients with a combination of other autoimmune diseases need to be excluded
the criteria for the classification of RA published jointly by the EULAR/American College of Rheumatology (ACR) in 2010	Patients with other underlying diseases, such as malignant tumors, hematologic diseases, endocrine and metabolic diseases, and severe infections, should be excluded
2016 ACR-EULAR Classification Criteria for primary Sjögren's Syndrome	Women who are breastfeeding or pregnant should be excluded
Complete clinical information, including laboratory results	Incomplete clinical treatment
